# Beyond Individual Risk: Neighborhood Effects on Loneliness in Predominately Black Adults in Pittsburgh

**DOI:** 10.1093/geroni/igaf122.4361

**Published:** 2025-12-31

**Authors:** Moka Yoo-Jeong, Greta Jianjia Cheng, Tamara Dubowitz, Wendy Troxel, Gerald Hunter, Bonnie Dastidar, Tanisha Hill-Jarrett, Andrea Rosso

**Affiliations:** Northeastern University, Boston, Massachusetts, United States; University of Pittsburgh, Pittsburgh, Pennsylvania, United States; University of Pittsburgh School of Public Health, Pittsburgh, Pennsylvania, United States; RAND, Pittsburgh, Pennsylvania, United States; Rand Corporation, Pittsburgh, Pennsylvania, United States; RAND, Santa Monia, California, United States; University of California Memory and Aging Center, San Francisco, California, United States; University of Pittsburgh, Pittsburgh, Pennsylvania, United States

## Abstract

While individual correlates (age, sex, physical function) of loneliness have been well-documented, less is known about whether neighborhood characteristics modify these associations. Guided by the Ecological Theory of Aging, this study examined whether subjective and objective neighborhood factors moderate the associations between individual correlates and loneliness among adults in two predominantly Black neighborhoods. Data were drawn from the Think PHRESH study (N = 753), an ancillary study to a longitudinal cohort study of the Pittsburgh Hill/Homewood Research on Neighborhood Change and Health (PHRESH). Loneliness was measured using the UCLA 3-item scale. Subjective neighborhood factors included perceived safety and neighborhood satisfaction. Objective factors included socioeconomic deprivation and residential instability. Multivariable linear regressions with interaction terms tested moderation effects. Among those perceiving their neighborhoods as less safe, middle-aged adults (50-65 years) and older adults (≥65 years) reported significantly higher loneliness compared to younger adults (31-49 years). These age-related differences were not observed among those reporting high safety. A similar pattern emerged for neighborhood satisfaction: greater loneliness was reported among middle-aged and older adults with low neighborhood satisfaction, but not among those with high satisfaction. Functional limitations were also linked to greater loneliness among those with low perceived safety or neighborhood satisfaction. Objective neighborhood measures did not moderate associations. Findings demonstrate that perceptions of neighborhood safety and satisfaction interact with individual vulnerabilities to shape loneliness. Supportive neighborhood conditions may buffer loneliness among high-risk groups. These findings highlight the need for community-based interventions that enhance subjective neighborhood experiences to buffer against loneliness in historically marginalized communities.

